# A nuclear export signal within the structural Gag protein is required for prototype foamy virus replication

**DOI:** 10.1186/1742-4690-8-6

**Published:** 2011-01-21

**Authors:** Noémie Renault, Joelle Tobaly-Tapiero, Joris Paris, Marie-Lou Giron, Audrey Coiffic, Philippe Roingeard, Ali Saïb

**Affiliations:** 1 CNRS UMR7212, Inserm U944, Université Paris Diderot, Institut Universitaire d'Hématologie, Paris, France; 2 Université François Rabelais- Inserm U966, Tours, France; 3 Conservatoire National des Arts et Métiers, Paris, France

## Abstract

**Background:**

The Gag polyproteins play distinct roles during the replication cycle of retroviruses, hijacking many cellular machineries to fulfill them. In the case of the prototype foamy virus (PFV), Gag structural proteins undergo transient nuclear trafficking after their synthesis, returning back to the cytoplasm for capsid assembly and virus egress. The functional role of this nuclear stage as well as the molecular mechanism(s) responsible for Gag nuclear export are not understood.

**Results:**

We have identified a leptomycin B (LMB)-sensitive nuclear export sequence (NES) within the N-terminus of PFV Gag that is absolutely required for the completion of late stages of virus replication. Point mutations of conserved residues within this motif lead to nuclear redistribution of Gag, preventing subsequent virus egress. We have shown that a NES-defective PFV Gag acts as a dominant negative mutant by sequestrating its wild-type counterpart in the nucleus. Trans-complementation experiments with the heterologous NES of HIV-1 Rev allow the cytoplasmic redistribution of FV Gag, but fail to restore infectivity.

**Conclusions:**

PFV Gag-Gag interactions are finely tuned in the cytoplasm to regulate their functions, capsid assembly, and virus release. In the nucleus, we have shown Gag-Gag interactions which could be involved in the nuclear export of Gag and viral RNA. We propose that nuclear export of unspliced and partially spliced PFV RNAs relies on two complementary mechanisms, which take place successively during the replication cycle.

## Introduction

Retroviral Gag proteins are involved in early stages of infection such as trafficking of incoming viruses and nuclear import (reviewed in [1]). Additionally, during the late phases of infection, they coordinate the assembly of viral particles, selecting the viral genome for encapsidation and directing the incorporation of the envelope glycoproteins [2]. For most retroviruses, expression of Gag alone is sufficient to induce the formation and release of virus like particles. For that purpose, retroviruses hijack the cellular endosomal machinery, enrolling components of the class E vacuolar protein sorting (VPS) machinery that induce topologically analogous membrane fission events [3,4]. In addition to these defined assembly domains, independent subcellular trafficking and/or retention signals that provide important functions in the virus life cycle have been identified (for a review, see [5]).

Foamy viruses (FVs) are complex exogenous animal retroviruses that differ in many aspects of their life cycle from orthoretroviruses such as the human immunodeficiency viruses (HIV) [6]. For example, Gag and Pol proteins of FVs are expressed independently of one another [7], and both proteins undergo a single cleavage event [8]. Hence, the structural Gag protein is not cleaved into the matrix, capsid, nucleocapsid sub-units as in most retroviruses, but is C-terminally cleaved by the viral protease, leading to the production of a Gag doublet during viral replication. Moreover, FV Gag is not myristoylated, and none of the conventional Gag landmarks of exogenous retroviruses, such as the major homology region or Cys-His motifs, are found in this protein [6]. Instead, prototype foamy virus (PFV) Gag harbors conserved C-terminal basic motifs, referred to as Gly-Arg (GR) boxes [9]. Although the first GR (GRI) box binds viral nucleic acids and is required for viral genome packaging [10], the second (GRII) harbors a nuclear localization sequence (NLS) at its C-terminus, targeting Gag to the nucleus early after infection [7,11]. Although this NLS is not absolutely required for productive infection, since other NLSs in Pol are likely involved in nuclear import of pre-integration complexes [12], it determines multiple integration events [13]. GRII also contains a chromatin binding sequence (CBS) in its N-terminus, tethering the PFV incoming pre-integration complex onto host chromosomes prior to integration [14]. Therefore, depending upon the stage of the viral cycle and thanks to these motifs, PFV Gag harbors distinct sub-cellular localizations. Of note, PFV does not encode a post-transcriptional regulator such as Rev or Rex from HIV or HTLV, respectively [15]; and therefore the mechanisms responsible for nuclear export of singly spliced or unspliced viral mRNA, such as the one encoding for the structural Gag proteins, are still not known. Similarly, where in the infected cell Gag initially interacts with the viral genome, is not known.

Similar to Mason-Pfizer monkey virus (MPMV) [16], PFV assembles into capsids intracellularly at a pericentriolar site [17]. Cytoplasmic PFV capsid assembly, which only requires the expression of Gag proteins, as for other retroviruses, is mediated by a motif akin to a cytoplasmic targeting and retention signal (CTRS) [18], also found in MPMV Gag [19]. Both domains harbor a conserved and indispensable arginine residue. However, unlike MPMV, budding of PFV is absolutely dependent upon the presence of cognate Env protein, implying a specific interaction between the Gag and Env proteins that may occur at the trans-Golgi network [17]. The unusually long leader peptide of PFV Env is likely involved in this specific interaction with the respective Gag domains located in the N-terminus of the protein, which are distinct from the CTRS [20]. Finally, PFV Gag was shown to interact with components of the VPS machinery for virus egress [21-23].

During viral replication, PFV Gag shows distinct sub-cellular localizations. During early stages of infection, incoming Gag can be found near the microtubule-organizing center (MTOC) and in the nucleus [24,25], similar to incoming HIV-1 Gag [26]. During the late stages of infection, following its synthesis in the cytoplasm, PFV Gag displays a transient nuclear localization triggered by the NLS present within its C-terminus [11]. Since PFV capsid assembly occurs near the centrosome [17] and the presence of Gag is required for Pol packaging [10], nuclear export of Gag is an absolute prerequisite for the completion of the retroviral cycle. The role of this nuclear stage as well as the molecular mechanism(s) responsible for nuclear export of PFV Gag are not yet understood.

Although this transient nuclear localization was initially thought to be a specific feature of PFV, other retroviral Gag proteins were shown to display a similar distribution during the late stages of infection. This is the case for example for HIV-1 [27] or Rous Sarcoma Virus (RSV) [28] Gag. For RSV, the nuclear stage of Gag proteins contributes to viral genomic RNA packaging [29], while the exact role of nuclear Gag is not clear in the case of HIV-1. Remarkably, both Gag proteins harbor a short hydrophobic motif that actively directs their nuclear export [27,28]. These so called leucine-rich nuclear export signals (NES) are recognized by exportin 1, also named CRM1, a member of the β importin superfamily of soluble nuclear transport receptors (reviewed in [30,31]). The first viral ligand of CRM1 identified was the HIV-1 Rev protein, which serves as an adaptor for the export of the unspliced and singly spliced viral mRNA that would otherwise be restricted from leaving the nucleus [32]. Leptomycin B (LMB) binds specifically to the central domain of CRM1, preventing interaction with the NES and inhibiting subsequent nuclear export [33-35].

Here, we identify a LMB-sensitive nuclear export sequence within the N-terminus of the PFV Gag. Point mutations of residues conserved among primate foamy viruses enhance nuclear distribution of the corresponding Gag mutants. Consequently, recombinant viruses produced in the presence of NES-defective Gag mutants were non-infectious. NES-defective Gag proteins behave as dominant negative mutants over their wild-type counterpart, preventing viral particle release. Finally, substituting the LMB-sensitive NES of PFV Gag with that of HIV-1 Rev lead to nucleocytoplasmic redistribution of the chimeric Gag protein, but failed to restore infectivity.

## Methods

### Cells and drugs

HeLa and 293T cells were cultured in Dulbecco's modified Eagles's medium supplemented with 10% fetal bovine serum, 2 mM L-glutamine, 20 mM Hepes and antibiotics (1% penicillin and streptomycin). Leptomycin B (LMB) (Sigma) was added to culture medium of transfected cells to a final concentration of 40 nM for 6 hours.

### Vector production

Vector stocks were produced by transfection of 293T cells using Polyfect (Qiagen) with equimolar quantity of the PFV pMD9 vector together with Gag (pCZIgag4), Pol (pCZIpol1) and Env (pCZHFVenvEM02) expressing plasmids kindly provided by A. Rethwilm [36]. Twenty-four hours post-transfection, CMV promoter transcription was enhanced by addition of 10 mM of sodium butyrate for 6 h. Twenty-four hours later, supernatants were clarified, filtrated through 0.45-μm-pore-size filters, concentrated by centrifugation on filter Amicon (Millipore) and conserved at - 80°C until use.

### Viral stocks titration

Infectious titers were determined by transduction of 293T cells with dilutions of vector stocks by spinoculation at 1,200 *g *for 1 h 30 minutes at 30°C. Forty-eight hours later, the cells were harvested and fixed in 1% paraformaldehyde (PFA), and the amounts of GFP-positive cells were determined by fluorescence-activated cell sorting on a FACScan device with CellQuest software (Becton Dickinson). The titer was calculated as follows: *T *= (*F *x*C*/*V*)x*D *(*F *is the frequency of GFP-positive cells, *C *is the number of cells at the time of infection, *V *is the volume of the inoculum, and *D *is the factor of dilution), expressed as transducing units (tu)/milliliter.

### Constructs

The full-length green fluorescent protein (GFP)-Gag expression plasmid (pGFP-Gag) was previously described [24]. Concerning Gag-RevNES, amino acids 95 to 112 were substituted by the 11aa of the HIV-1 RevNES in pCZIgag4 by two-steps procedure: deletion of aa 95-112 to generate GagΔ95-112 and then insertion of 11aa of RevNES to obtain Gag-RevNES. The GFP-NES expression plasmids were generated by inserting the annealing products of appropriate complementary oligonucleotides into the SacI-EcoRI sites of the pEGFP-C3 vector (Clontech). The tagged His-HA Gag expression plasmid, pCZIGagPGCLHH (noted as GagHH), was kindly provided by D. Lindemann. Mutations of the different expression plasmids were created using the QuickChange site-directed mutagenesis protocol according to the manufacturer's specifications (Stratagene). All PCR-generated clones were confirmed by sequencing. Primer sequences are available upon request.

### Immunocytochemistry

Cells, grown on glass coverslips, were transfected with wild-type expression plasmids or derived mutants using Polyfect reagent (Qiagen). Twenty-four hours post-transfection, the cells were rinsed with phosphate-buffered saline (PBS), fixed with 4% PFA for 15 minutes at 4°C, and permeabilized with methanol for 5 minutes at 4°C. After blocking (0.1% Tween 20, 3% bovine serum albumin in PBS), coverslips were successively incubated with mouse monoclonal anti-HA 12CA5 (Roche) serum overnight at 4°C (1/2000). Cells were then washed and incubated for 30 min with a 1/800 dilution of the appropriate fluorescent-labeled secondary antibody. Finally, nuclei were stained with 4,6-diamidino-2-phenylindole (DAPI), and the coverslips were mounted in Moviol. Confocal microscopy observations were performed with a laser-scanning confocal microscope (LSM510 Meta; Carl Zeiss) equipped with an Axiovert 200 M inverted microscope, using a Plan Apo 63_/1.4-N oil immersion objective.

### Immunoprecipitation and Western blotting

Cells were lysed in Chaps buffer (10 mM Tris, pH 7.4, 0.15M NaCl, 0.1% (3cholamidopropyl)-dimethylamonio]-1-propanesulfonate (Chaps) in the presence of 1 mM Protease Inhibitor Cocktail (Roche) for 30 min 4°C. Cells lysates were centrifuged at 12,000 g for 5 min (supernatant: cytoplasmic fraction). Pelleted nuclei were lysed in Chaps buffer containing 0.85M NaCl (nuclear fraction). For co-immunoprecipitation experiments, cytoplasmic and nuclear fractions were incubated overnight at 4°C with anti-HA or anti-GFP mouse monoclonal antibodies (Roche), captured on protein A Sepharose (GE Healthcare), after 20 min treatment with 1.6 μg/ml cytochalasine D (Sigma). Immune complexes were washed 4 times with 0.85M NaCl Chaps lysis buffer and solubilised in Laemmli buffer.

Western-blotting was performed as follows: Samples were migrated on a SDS-10% polyacrylamide gel, proteins were transferred onto cellulose nitrate membrane (Optitran BA-S83; Schleicher-Schuell), and incubated with appropriate antibodies before being detected by enhanced chemoluminescence (Amersham). Rabbit polyclonal anti-PFV Gag, rabbit polyclonal anti-actin (Sigma), and mouse monoclonal anti-LDH (Sigma) were used.

### Electron microscopy

For electron miscroscopy (EM), transfected 293T cells were fixed *in situ *by incubation for 48 h in 4% paraformaldehyde and 1% glutaraldehyde in 0.1 M phosphate buffer (pH 7.2), and were then post-fixed by incubation for 1 h with 2% osmium tetroxide (Electron Microscopy Science, Hatfield, PA). They were dehydrated in a graded ethanol series, cleared in propylene oxyde, and then embedded in Epon resin (Sigma), which was allowed to polymerize for 48 h at 60°C. Ultrathin sections were cut, stained with 5% uranyl acetate 5% lead citrate, and then placed on EM grids coated with collodion membrane. They were then observed with a Jeol 1010 transmission electron microscope (Tokyo, Japan).

## Results

### A point mutation in the N-terminus of Gag inhibits capsid assembly and virus egress

**Figure 1:**
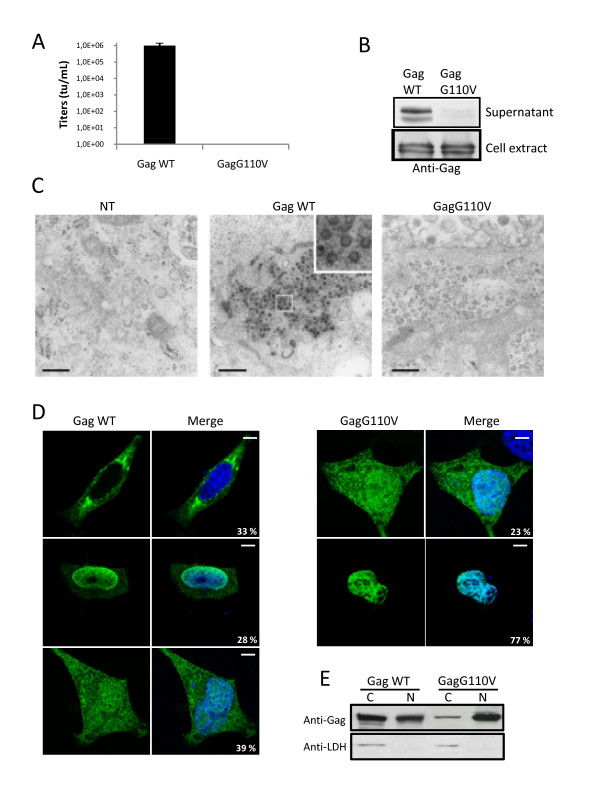
**Characterization of the GagG110V mutant**. **(A) **Transduction rate of viruses harboring either GagWT or GagG110V. 293T cells were transfected for 48 h with FV vector encoding for GFP together with plasmids expressing Env, Pol and GagWT or GagG110V. Cell free supernatants were used to transduce 293T cells and the viral titer was determined from the number of GFP-positive cells by FACS analysis 48 h post-transduction. No infectivity was detected in the supernatant of GagG110V transfected cells, as observed in five independent experiments. **(B) **Western blotting performed on 293T cellular extracts and cell free supernatants shows the absence of viral particles in the supernatant of GagG110V transfected cells whereas intracellular GagG110V is normally produced. **(C) **Electron microscopy revealed, furthermore, the absence of intracellular capsids in 293T cells transfected with GagG110V. Bar: 0.5 μm. **(D) **Subcellular localization of GagWT and GagG110V in Hela transfected cells with GagWT or GagG110V and analyzed, 24 h post-transfection, by confocal microscopy following indirect immunofluorescence using rabbit polyclonal anti-PFV. GagWT is either nucleocytoplasmic, cytoplasmic or nuclear whereas GagG110V is mainly nuclear, as observed in three independent experiments (approximately 200 cells were counted in each preparation). **(E) **Western blotting performed on fractionated Hela cell extracts of Gag WT and GagG110V. Detection of the human lactate dehydrogenase (LDH) in cytoplasmic extracts only attests the validity of the fractionation assay (C: Cytoplasm, N: Nucleus).

To decipher the implication of highly conserved residues among PFV Gag proteins on the sub-cellular localizations of this structural protein and their respective roles during viral replication, a series of point mutations was introduced into the N-terminus part of the protein. The corresponding Gag constructs were used to produce PFV-derived recombinant viruses in a vector system as already reported [37]. Briefly, 293T cells were transfected with a GFP encoding PFV-derived vector together with homologous Pol, Env and Gag expression plasmids. Twenty-four hours post-transfection, cell-free supernatants were used to transduce 293T cells, and the remaining transfected cells were lysed for Western-blotting analysis. Forty-eight hours post-transduction, GFP expression was monitored by flow cytometry. The use of the wild-type (WT) Gag expressing plasmid led to efficient production of infectious recombinant viruses. In contrast, when a Gag mutant harboring a glycine to valine substitution at position 110 (GagG110V) was transfected instead of its wild-type counterpart, GFP positive cells were not detected by FACS following transduction (Figure 1A). Western-blot analysis of the corresponding cell-free supernatant demonstrated the absence of the characteristic 71/68 kDa Gag doublet, whereas intracellular Gag proteins, efficiently cleaved, were similarly detected in both producer cells (Figure 1B). These observations demonstrate that the G110V substitution does not impair expression and processing of the Gag polyprotein, but precludes virus production.

Lack of virus production could either be due to impairment of virus release due to a Gag-Env interaction defect and/or capsid assembly deficiency. Since it was reported that the Gag domain involved in Gag-Env interaction is located upstream of residue 92 [36], the second hypothesis was assessed. For that purpose, electron microscopy analysis was performed on 293T cells transfected with either wild-type Gag or GagG110V expressing plasmids. As shown in figure 1C, normal shaped viral capsids were easily detected in the cytoplasm from cells transfected with wild-type Gag. In contrast, no viral capsid was observed in cell cultures transfected with a GagG110V expressing plasmid. Therefore, the G110V substitution prevents capsid assembly, impairing subsequent virus egress.

### The GagG110V mutant is restricted to the nucleus

To understand the molecular basis of the defect in capsid assembly observed with the GagG110V mutant, its sub-cellular localization was analyzed in transfected Hela cells in comparison with its wild-type counterpart. Twenty-four hours post-transfection with wild-type or mutated Gag expressing plasmids, cells were fixed, permeabilized and Gag proteins were stained for indirect immunofluorescence using anti-Gag antibodies. Wild-type Gag proteins were detected in the cytoplasm for 33% ± 2% of transfected cells, including around the centrosome, within the nucleus (28% ± 2%) or harbored a nucleocytoplasmic distribution (39% ± 2%) (Figure 1D). Conversely, GagG110V was mainly confined in the nucleus (77% ± 2% of transfected cells), some GagG110V-positive cells exhibiting a nucleocytoplasmic staining (23% ± 2% of transfected cells) (Figure 1D). These sub-cellular localizations were confirmed by western-blot following cell fractionation (Figure 1E). Note that wild-type Gag and GagG110V were similarly maturated by viral protease (see Figure 1B). Moreover, electron microscopy analysis of GagG110V transfected cells did not reveal any Gag-derived nuclear structures (Figure 1C).

Several hypotheses could explain this observation. (i) First, the G110V mutation could lead to a conformational change which efficiently exposes the GRII NLS, dominantly targeting the mutant protein in the nucleus. (ii) In addition, this mutation could also unmask a cryptic NLS in the N-terminus that may synergize with the GRII NLS. (iii) This mutation could also create a second nuclear retention motif, the first one being the CBS in GRII [14], trapping more efficiently Gag in the nuclear compartment. (iv) This mutation could indirectly affect a region necessary to maintain Gag in the cytoplasm, such as the CTRS. (v) Finally, the G110V substitution could affect a nuclear export signal that allows cytoplasmic redistribution of Gag following its nuclear import.

### The G110 is part of a leucine rich nuclear export motif

**Figure 2:**
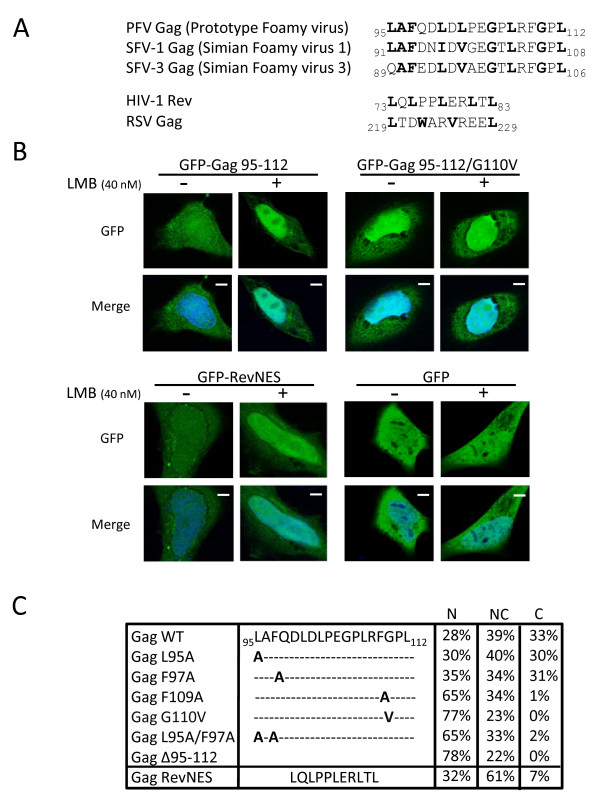
**Identification of a functional NES in PFV Gag**. **(A) **Sequence alignment of a N-terminal region within Gag protein of primate foamy viruses. **(B) **Subcellular localization of GFP-Gag 95-112 and derived G110V mutant in Hela cells in the presence or the absence of LMB (40nM). GFP-RevNES and GFP alone were used respectively as positive and negative controls. Representative fluorescence images of the vast majority of cells expressing the indicated fusion proteins are shown by confocal microscopy. **(C) **Amino acid(s) important for Gag nuclear export. Point mutations or deletion were generated in the context of full length Gag and the resulting mutants were tested for sub-cellular localization after 24 h transfection using rabbit polyclonal anti-PFV antibodies. Results concerning Gag-RevNES localization were included. The numbers shown are the means of three independent experiments by counting 200 cells each (N: nuclear, NC: nucleocytoplasmic, C: cytoplasmic localization).

Interestingly, the G110 amino-acid is located within a stretch of conserved hydrophobic residues, between aa 95 and 112 (Figure 2A), that is predicted to constitute a leucine-rich NES by the NetNES Prediction method [38]. To directly assess the last assumption, amino acids 95 to 112 from PFV Gag was cloned in frame to the C-terminus of the green fluorescent protein (GFP-Gag 95-112) and the sub-cellular localization of the corresponding fusion protein was analyzed following transfection of Hela cells in the presence or absence of leptomycin B (LMB), a specific inhibitor of the CRM1-dependent nuclear export pathway. The prototypic NES of HIV-1 Rev, fused to the C-terminus of GFP (GFP-RevNES), was used as a positive control. As shown in figure 2B, GFP-RevNES showed a nucleocytoplasmic distribution in the absence of LMB, probably due to passive diffusion through the nuclear pores. As expected, under LMB treatment, GFP-RevNES concentrated in the nucleus. A nucleocytoplasmic distribution was also observed for GFP-Gag 95-112 in the absence of LMB. Remarkably, GFP-Gag 95-112 mainly concentrated in the nucleus following LMB treatment. In the context of GFP-Gag 95-112, the G110V mutation led to a nuclear localization of the corresponding mutant, with or without LMB treatment. Note that the sub-cellular distribution of wild-type GFP alone, used as negative control, was not affected by LMB treatment (Figure 2B).

Furthermore, deleting amino acids 95 to 112 on the full length PFV Gag, and to a lesser extent, point mutations of conserved residues, led to nuclear redistribution of the corresponding mutants (Figure 2C). GagF109A, GagL95A/F97A and GagΔ95-112 mutants, which each showed a similar distribution as the G110V mutant, were further examined for release particle and infectivity (data not shown) and behaved as G110V (see Figure 1).

Therefore, the PFV Gag domain encompassing aa 95 to 112 constitutes an effective LMB-sensitive nuclear export signal. This sequence will be referred to the Gag NES. Consequently, the lack of viral capsids in GagG110V transfected cells relies on efficient nuclear confinement of the mutant proteins.

### The GagG110V mutant harbors dominant negative properties by sequestrating wild-type Gag in the nucleus

**Figure 3:**
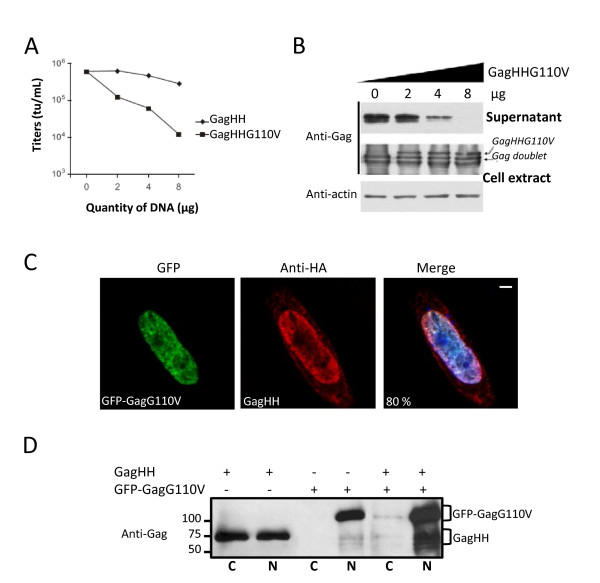
**Dominant-negative properties of the GagG110V mutant**. **(A) **Virus titers. Viral particles were produced in the supernatant of 293T cells transfected with the four-plasmid PFV vector system in the presence of increasing amounts of GagHH or GagHHG110V. Target 293T cells were transduced with cell free supernatants and titers were determined by FACS analysis 48 h post-transduction. Viral titers were dramatically reduced following addition of increasing amounts of GagHHG110V. This result is representative of three independent experiments. **(B) **Western blotting also shows a decrease in the amount of Gag proteins in supernatants whereas they are efficiently produced in 293T cells extracts. Therefore, GagG110V mutant negatively interferes with WT Gag impairing particles production. **(C) **Co-localization of GagHH and GFP-GagG110V. Hela cells were co-transfected with indicated plasmids and analyzed, 48 h post-transfection, by confocal microscopy following indirect immunofluorescence. GagWT colocalizes with GFP-GagG110V in the nucleus in 80 ± 4% of transfected cells in three independent experiments with approximately 100 cells counted each time. **(D) **Sequestration of GagWT by GagG110V in the nucleus. Nuclear interaction of GagHH and GFP-GagG110V revealed by co-immunoprecipitation of nuclear extracts of transfected Hela cells, using mouse anti-HA or anti-GFP antibodies followed by western-blotting performed with rabbit polyclonal anti-Gag antibodies (N : nucleus and C : cytoplasm).

We then asked whether a NES-defective Gag mutant could negatively interfere with the replication of wild-type PFV Gag. This was assessed by quantifying recombinant virus production in the presence or in the absence of a NES-defective Gag mutant in the same system as the one used in figure 1. PFV-derived vector encoding for GFP, together with Pol, Gag and Env expressing plasmids were transfected in 293T cells. Increasing amounts of either wild-type Gag or a GagG110V expressing plasmids were co-transfected in parallel experiments. For this experiment, we used a GagG110V expressing plasmid in which the Gag open reading frame was fused to the His and HA tags (named GagHHG110V), since its presence was easily detected in cell extracts as a higher molecular size band by Western-blot. Forty-eight hours later, cell free supernatants were collected and viral titers were evaluated by FACS following transduction of 293T cells. Whereas co-transfection of the wild-type GagHH plasmid had only a minor effect on virus production, the presence of GagHHG110V impaired virus release in a dose dependent manner (Figure 3A). Biochemical analysis confirmed this observation since the 71/68 kDa Gag doublet in cell-free supernatants decreased concomitantly with increasing amounts of GagHHG110V, the latter was detected as a higher molecular band (Figure 3B). Note that similar to GagG110V, GagHHG110V was never detected in cell free supernatants (Figure 3B). These results demonstrated that the NES-defective Gag mutant dominantly interferes with viral particle release.

Since PFV Gag-Gag interactions were demonstrated in the nucleus [39] and given that GagG110V is mainly confined in the nucleus, we wondered whether the dominant negative effect of the GagG110V protein relies on nuclear retention of wild-type Gag proteins via intranuclear Gag-Gag interactions. To substantiate this, HeLa cells were transfected with wild-type Gag fused with His and HA tags (GagHH) and GFP-GagG110V expression plasmids, and their respective sub-cellular localizations were studied by indirect immunofluorescence followed by confocal analysis, forty-eight hours post-transfection. Whereas wild-type Gag expressed alone showed distinct localizations (data not shown), as previously reported (Figure 1D), it was mainly restricted in the nucleus in the presence of GFP-GagG110V (80% ± 4% of transfected cells, Figure 3C). These observations were confirmed at the biochemical level by co-immunoprecipitation assays. Whereas wild-type Gag was detected in both the nuclear and cytoplasmic fractions when expressed alone, it was mainly restricted in the nucleus when co-expressed with GFP-GagHHG110V (Figure 3D).

Altogether, these results demonstrated that the dominant negative property of GagG110V mainly relies on nuclear retention of wild-type Gag, precluding Gag nuclear export and subsequent capsid assembly.

### The NES of HIV-1 Rev could only partially trans-complement that of PFV Gag

**Figure 4:**
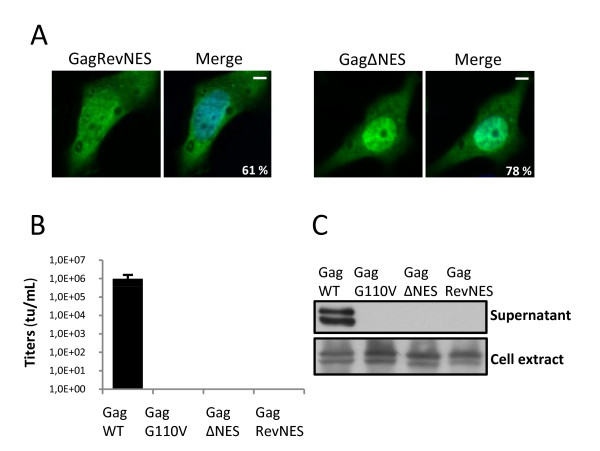
**HIV-1-RevNES fails to restore infectivity**. **(A)** Subcellular localization of GagΔNES and Gag-RevNES in Hela cells analyzed 24h post-transfection with PFV antibodies by confocal microscopy following indirect immunofluorescence in three independent experiments (approximately 200 cells counted each time). **(B) **Transduction rate of viruses harboring either GagWT, GagG110V, GagΔNES or Gag-RevNES. Cell free supernatants were used to transduce 293T cells and the viral titer was determined from the number of GFP-positive cells by FACS analysis 48h post-transduction. No infectivity was detected in the supernatants of GagG110V, GagΔNES and Gag-RevNES transfected cells in four independent experiments. **(C) **Western blotting performed on 293T cell extracts and cell-free supernatants shows the absence of viral particles in the supernatants of GagG110V, GagΔNES and Gag-RevNES transfected cells whereas the intracellular Gag mutants are normally produced and matured.

To assess whether an heterologous LMB-sensitive NES could functionally trans-complement that of PFV Gag, the latter was replaced by the NES of HIV-1 Rev. The resulting Gag chimeric construct, named Gag-RevNES, was transfected in 293T cells, and its sub-cellular localization was analyzed. As shown in figure 4A and 2C, Gag-RevNES displayed a predominant nucleocytoplasmic distribution (61% ± 2%). As a control, GagΔNES was mainly detected in the nucleus (78% ± 2% of transfected cells). Since the NES of HIV-1 Rev was shown to restore the nucleocytoplasmic distribution of PFV Gag, we next assessed whether the chimeric Gag protein was able to restore infectivity of recombinant viruses. For that purpose, wild-type Gag, GagG110V, GagΔNES or Gag-RevNES expressing plasmids were used to produce recombinant viruses following transfection of 293T cells with a GFP expressing PFV vector together with Env and Pol expressing plasmids. Forty-eight hours post-transfection, cell-free supernatant was used to transduce 293T cells, and GFP expression was monitored by flow cytometry forty-eight hours later. Remarkably, only the use of wild-type Gag led to the production of infectious viruses (Figure 4B). Western-blot analysis of cell-free supernatants from transfected 293T cells demonstrated the presence of the Gag doublet when wild-type Gag was used and their absence when using GagG110V or GagΔNES to produce recombinant viruses, as expected. Importantly, no Gag doublet was detected when using the Gag-RevNES construct, whereas these proteins were efficiently expressed in producer cells (Figure 4C). These results demonstrated that the heterologous leucine rich NES of HIV-1 Rev, which allowed efficient nucleocytoplasmic redistribution of PFV Gag deleted from its own NES, failed to restore infectivity of the corresponding recombinant viruses.

## Discussion

The late occurring nuclear targeting of Gag proteins, which was initially thought to be a specific feature of PFV [11], was also demonstrated for distinct retroviruses, such as Rous Sarcoma Virus (RSV) [28] and also for the retrotransposon Tf1 [40]. Hence, for RSV and PFV, following proviral integration, the late stages of infection can be divided into an early (synthesis of Gag and its nuclear translocation) and late (nuclear export of Gag, capsid assembly and virus egress) phases [41]. We show here that nuclear export of PFV Gag proteins relies on a LMB-sensitive leucine-rich nuclear export sequence (NES) within the N-terminus of the structural protein. NES-defective Gag proteins are mainly located in the nucleus when compared to their wild-type counterpart. Using NES-defective Gag mutants, production of PFV-derived recombinant viruses was unsuccessful, their nuclear localization preventing the formation of viral capsids in the cytoplasm and subsequent virus egress. Moreover, NES-defective Gag proteins behave as dominant negative (DN) mutants by sequestrating wild-type Gag in the nuclear compartment. This DN effect is reminiscent to what has been already reported in the case of DN mutants for HIV-1 Rev [42-44] or for HIV-1 Gag [45]. Note that the sub-cellular distribution of a chimeric PFV Gag protein, in which the NES of Gag was replaced with that of HIV-1 Rev, efficiently induces the nucleocytoplasmic redistribution of the fusion protein. Remarkably, no extracellular virus was detected when the Gag chimera was used instead of its wild-type counterpart for the production of PFV-derived recombinant viruses (Figure 4C). This substitution could alter the tridimensional structure of PFV Gag, preventing essential Env-Gag interactions required for virus egress. Alternatively but not exclusively, nuclear export driven by the NES of HIV-1 may trigger a cytoplasmic localization of the chimeric Gag protein distinct from that of its wild-type counterpart, preventing subsequent late stages of the viral cycle.

Sequential dimerization, oligomerization, and multimerization of Gag proteins are finely tuned to regulate their functions, in particular for proper capsid assembly and subsequent virus release [1]. PFV Gag-Gag interactions mainly occur via distinct motifs along this polyprotein [36,46], including a coiled-coil domain (called CC2) located in the N-terminal part [39]. We show here that a NES-defective Gag could retain its wild-type counterpart, in the nucleus, confirming the existence of Gag-Gag interactions in this compartment, as recently demonstrated for RSV Gag [41]. These results are consistent with our previous observations. Indeed, when PFV Gag was fused to the promyelocytic leukemia protein (PML), the chimera was restricted onto PML-nuclear bodies (NBs), structures belonging to the nuclear matrix [39]. When wild-type Gag, but not a CC2-deleted mutant which was defective for Gag-Gag interaction, was expressed in these cells, it delocalized the PML-Gag fusion from NBs to a diffuse but nuclear staining, demonstrating the existence of nuclear Gag-Gag interactions. These nuclear interactions were demonstrated also at the biochemical level by co-immunoprecipitation. Of course, this does not exclude the existence of interactions that could take place in the cytoplasm, as is also the case for RSV Gag [41].

What is the role of PFV Gag nuclear stage? In higher eukaryotic cells, pre-mRNAs are retained in the nucleus until they are fully spliced (for a review [47]). Therefore, to overcome this quality control, retroviruses have developed different strategies to export their unspliced or partly spliced mRNAs, hijacking cellular nuclear export machineries (reviewed in [48]). Simple retroviruses generally harbor *cis*-acting sequences involved in viral RNA nuclear export [49]. In contrast, in most of complex retroviruses, small regulatory proteins deal with this cellular restriction. For example, HIV-1 encodes Rev, a nucleocytoplasmic shuttling protein that bridges unspliced and incompletely spliced viral RNAs on the Rev-responsive element (RRE) -a *cis*-acting element located within the *env *gene- to CRM1, thanks to its leucine-rich nuclear export sequence [32]. For the Jaagsiekte Sheep Retrovirus (JSRV), an unusually long Env leader peptide contributes to viral nuclear export [50]. PFV, although harboring a complex genomic organization, does not encode a functional Rev-like protein [15] and its Env leader peptide was not implicated in nuclear export but was shown to be involved in Env-Gag interactions required for virus budding [20].

In the case of RSV, Gag dimerization is promoted by binding to viral RNA, as already proposed for other retroviruses [51]. This, which mainly occurs in the nucleus, triggers a conformational change that unmasks an efficient NES within the p10 domain of the Gag polyprotein, resulting in nuclear export of Gag-RNA complexes [52,53]. Remarkably, prior to Gag synthesis, nuclear export of intron-containing RNA likely relies on *cis*-acting direct repeat sequences located in the 3' end of the viral genome, involving the cellular TAP/NXF1 and Dbp5 export factors [54]. The cytoplasmic fate of the viral genome could rely on the use of one of these two pathways, leading either to its packaging following Gag-dependent nuclear export or translation if based on *cis*-acting sequences. Indeed, there is a mechanistic link between retroviral RNA trafficking, in particular the way it is exported from the nucleus, and viral protein activities in the cytoplasm, affecting distinct late cytoplasmic stages such as capsid assembly, genome packaging and/or virus budding [49,55-58]. Of note, upon inclusion of Gag sequences from more distantly related FV species, such as the one from the feline isolate into the alignment, the C-terminal part contains a highly conserved short motif with the PFV Gag G110 residue being 100% conserved throughout. However, the Gag protein from the feline foamy virus (FeFV), although detected close to perinuclear regions, seems to be excluded from the nucleus [59]. Either nuclear export of FeFV Gag is extremely efficient and therefore the nuclear stage is not easily discernible or, alternatively during infection, other viral components are required for nuclear export of unspliced or partly spliced mRNAs.

**Figure 5:**
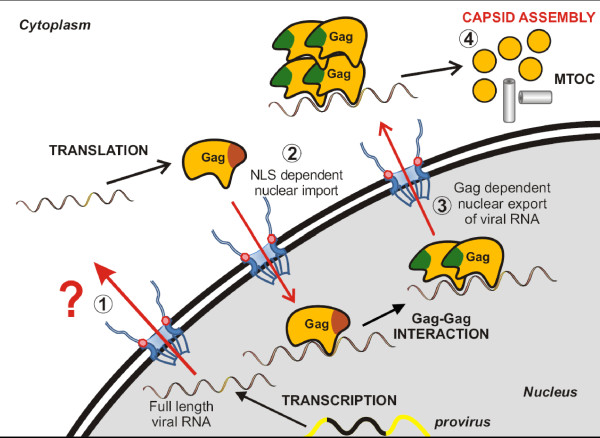
**Model for the possible nuclear role of FV Gag during the late stages of infection**. (1) Full length viral RNA export is still unknown. (2) After synthesis in the cytoplasm, Gag protein enters the nucleus via its NLS domain (located within the GRII box). In the nucleus, Gag could interact with the full length viral RNA via its GRI box favoring Gag-Gag interaction and subsequently unmasking Gag NES. (3) The nuclear export factor, CRM1, also called exportin 1, would then be able to interact with this ribonucleoprotein complex leading to its efficient nuclear export. (4) In the cytoplasm, Gag proteins will multimerize for capsid assembly near the MTOC. In the absence of Gag proteins, the initial nuclear export of unspliced PFV RNA could rely on another export mechanism independent of these proteins.

Therefore, based on our results, it would be interesting to assess whether PFV Gag proteins could be involved in this critical step, in a way similar to what was reported for RSV Gag. According to this model, PFV Gag proteins would bridge the nuclear intron-containing viral RNAs thanks to the GRI box to CRM1 via the leucine-rich NES we identified, promoting their nuclear export (Figure 5). In this context, PFV Gag proteins were effectively shown to interact with CRM1 in the presence of the PFV RNA packaging signal (preliminary results). Interaction between Gag and the viral RNA could occur either prior to Gag nuclear import or within the nucleus. In the cytoplasm, following nuclear export, Gag might transport viral RNAs towards the MTOC where capsid assembly and Pol packaging take place [17]. In a viral context, predominant nuclear localization of a PFV Gag protein deleted from its GR1 box [9], which was shown to be essential for viral nucleic acids binding, is in agreement with this working model.

Before Gag synthesis, initial nuclear export of intron-containing RNA could rely on *cis*-acting sequences on viral RNA, as already reported for RSV [54]. Remarkably, in that case, it seems that nuclear export is dependent on a structured RNA element and the cellular RNA-binding protein HuR as well as the adapter molecules ANP32A and B (pp32 and April) [60]. Thus, we propose that nuclear export of unspliced and partially spliced PFV RNAs relies on two complementary mechanisms, which take place successively during the replication cycle.

*Note added in proof: *Since the acceptation of this manuscript, the initial nuclear export pathway of mRNA PFV has been recently published online ahead of print on 15 December 2010 by Bodem J et al. [61].

## Competing interests

The authors declare that they have no competing interests.

## Authors' contributions

AS, NR, JTT conceived and designed the experiments; NR, JP, MLG, PR, AC, JTT performed the experiments; AS, MLG, JTT analyzed the data; AS wrote the manuscript.
